# Broad-Spectrum Antiviral Activity of RNA Interference against Four Genotypes of Japanese Encephalitis Virus Based on Single MicroRNA Polycistrons

**DOI:** 10.1371/journal.pone.0026304

**Published:** 2011-10-18

**Authors:** Zhiqiang Wu, Ying Xue, Bei Wang, Jiang Du, Qi Jin

**Affiliations:** State Key Laboratory for Molecular Virology and Genetic Engineering, Institute of Pathogen Biology, Chinese Academy of Medical Sciences and Peking Union Medical College, Beijing, People's Republic of China; University of Kansas Medical Center, United States of America

## Abstract

Japanese encephalitis virus (JEV), a neurotropic mosquito-borne flavivirus, causes acute viral encephalitis and neurologic disease with a high fatality rate in humans and a range of animals. Small interfering RNA (siRNA) is a powerful antiviral agent able to inhibit JEV replication. However, the high rate of genetic variability between JEV strains (of four confirmed genotypes, genotypes I, II, III and IV) hampers the broad-spectrum application of siRNAs, and mutations within the targeted sequences could facilitate JEV escape from RNA interference (RNAi)-mediated antiviral therapy. To improve the broad-spectrum application of siRNAs and prevent the generation of escape mutants, multiple siRNAs targeting conserved viral sequences need to be combined. In this study, using a siRNA expression vector based on the miR-155 backbone and promoted by RNA polymerase II, we initially identified nine siRNAs targeting highly conserved regions of seven JEV genes among strains of the four genotypes of JEV to effectively block the replication of the JEV vaccine strain SA14-14-2. Then, we constructed single microRNA-like polycistrons to simultaneously express these effective siRNAs under a single RNA polymerase II promoter. Finally, these single siRNAs or multiple siRNAs from the microRNA-like polycistrons showed effective anti-virus activity in genotype I and genotype III JEV wild type strains, which are the predominant genotypes of JEV in mainland China. The anti-JEV effect of these microRNA-like polycistrons was also predicted in other genotypes of JEV (genotypes II and IV), The inhibitory efficacy indicated that siRNAs×9 could theoretically inhibit the replication of JEV genotypes II and IV.

## Introduction

Japanese encephalitis virus (JEV), a neurotropic mosquito-borne flavivirus mainly prevalent in Asia, is the most important causative agent of acute viral encephalitis in humans [1]. It is estimated that JEV is responsible for at least 50,000 cases of viral encephalitis worldwide each year, resulting in 10,000–15,000 deaths and 15,000 cases with long-term, neuropsychiatric sequelae [2]. JEV is also one of the main causes of infectious reproductive failure in swine, resulting in significant economic losses in the pig industry. This virus has a normal zoonotic transmission cycle between swine or birds and mosquitoes. Swine are the main amplifier hosts, from which infected mosquitoes transmit the virus to humans [3]. Currently, a live attenuated JEV vaccine, SA 14-14-2, with excellent immunogenicity, is widely used in humans in China and other countries in Asia [2], [4]. Although great success has been achieved with this vaccine, there is still no specific therapy available for ongoing JEV infection in humans.

JEV is a member of the genus Flavivirus, part of the family Flaviviridae. The JEV genome is a positive single-stranded RNA molecule of about 11 kb in length. It contains a single open reading frame encoding a polyprotein that is flanked by 5′- and 3′- untranslated regions (UTRs). The polyprotein is co-translationally and post-translationally cleaved by the virus-encoded serine protease, NS2B/NS3, and by host-encoded proteases to produce three structural proteins (C, prM and E) and seven nonstructural proteins (NS1, NS2A, NS2B, NS3, NS4A, NS4B and NS5) [5], [6], [7]. Based on the nucleotide sequences of the C/PrM and E genes, JEV isolates have been classified into four genotypes (genotypes I, II, III and IV) and a postulated fifth genotype (Muar strain). Genotypes I and III are associated with epidemic regions in China, Korea, Japan, India, Thailand, the Philippines and other countries in Asia, whereas, genotypes II and IV are associated with endemic disease in Malaysia, Indonesia and northern Australia [8], [9], [10], [11], [12]. Among these, genotype III has the widest geographic distribution in Asia, and the vaccine strain, SA 14-14-2, is derived from wild type strain SA14 of genotype III.

RNA interference (RNAi), a mechanism widely present in eukaryotes, is an evolutionarily conserved process of sequence-specific gene silence initiated by double-stranded RNAs (dsRNAs) or microRNAs (miRNAs) [13]. In the case of dsRNAs, the dsRNAs are cleaved by Dicer (a host ribonuclease-III like enzyme) into 21–25 nt small interfering RNAs (siRNAs). These siRNAs are incorporated into a multi-protein complex known as “RNA-induced silencing complex” (RISC) in the cytoplasm and direct RISC to degrade homologous target mRNAs [14], [15]. In the case of miRNAs, the primary RNA transcripts (pri-miRNAs) are cleaved by Drosha into 60–70 nt miRNA precursors (pre-miRNAs) in the nucleus. Pre-miRNAs are transported into the cytoplasm by Exportin-5 and then cleaved into ∼22 nt miRNAs by Dicer. The mature miRNAs are incorporated into RISC and direct RISC to cleave mRNA or repress translation. Most pri-miRNA transcripts encode clusters of distinct miRNAs under the control of a single RNA polymerase II promoter [13], [16]. In mammalian cells, an RNAi effect can be induced by introducing either exogenous synthetic 21–29 nt siRNAs or RNA polymerase III-driven short-hairpin RNA (shRNA) vectors [17], [18], [19], [20].

In the past decade, RNAi has been used successfully for clearing mammalian cells of various viral infections [21], [22], [23], [24]. For JEV, a potent inhibitory effect has been reported with single siRNA or shRNA [3], [25], [26], [27]. However, because of the genetic variability of JEV [10], [11] and escape mutations in the targeted sequences [28], the therapeutic application of single siRNA or shRNA is limited. Therefore, multiple siRNAs targeting conserved viral sequences need to be combined in this case. Recently, a new miRNA-like siRNA expression platform based on miRNA principles has been reported in many studies. These siRNAs are expressed from the pri-miRNA backbone by constructing heterologous siRNAs into pri-miRNA hairpin stems under the control of a RNA polymerase II. Like most miRNAs that are transcribed from polycistronic pri-miRNAs, multiple siRNAs can be synthesized from a single polycistronic transcript using this strategy [22], [29], [30], [31], [32], [33]. This platform allows us to simultaneously transcribe to similar levels multiple, effective siRNAs against JEV from a single promoter-driven miRNA-like polycistronic construct.

In this study, we initially analyzed the molecular epidemiological characteristics of JEV, and determined the highly conserved sequences of eight JEV genes (PrM, NS1, NS2A, NS2B, NS3, NS4A, NS4B and NS5) among the four genotypes. Thirteen siRNAs against the selected conserved regions of these genes were designed and constructed individually into a RNA polymerase II-driven siRNA expression vector based on the miR-155 backbone. Using a siRNA-to-target reporter assay system [34], [35], nine effective siRNAs out of the 13 tested were selected to investigate their inhibitory effects on JEV replication in cultured cells. Then, these nine siRNA cassettes were combined in different sequences using the polycistronic nature of the miRNAs. The results showed that either a single siRNA or multiple siRNAs of these nine siRNAs could inhibit the replication of the genotype III strain, the live attenuated vaccine strain SA14-14-2. Finally, three wild type strains (SH53, SH101 and SA14) of genotypes I and III, which are predominant in mainland China, were chosen to validate the anti-JEV effect of these multiple siRNAs from miRNA-like polycistrons. The replication of the three wild type strains was inhibited effectively by these miRNA-like polycistrons. Furthermore, the anti-JEV effect of these miRNA-like polycistrons was predicted in genotypes II and IV, and the effect of a few nucleotide mismatches within the target regions was also estimated.

## Results

### Design and selection of siRNAs against JEV

**Figure 1:**
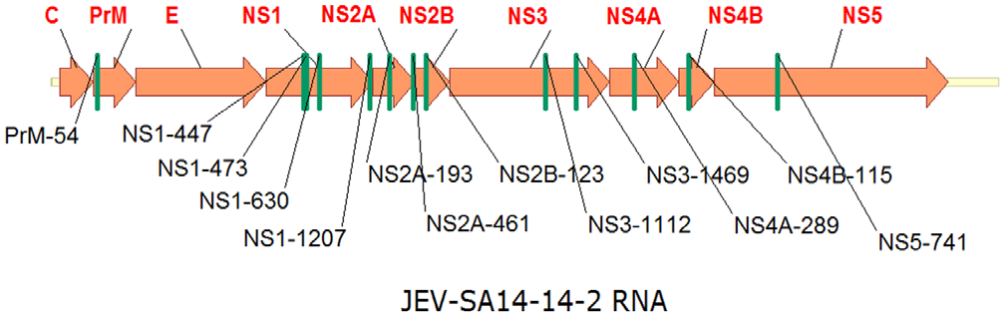
Schematic representations of the JEV RNA structure and the siRNA target sites. Thirteen target sites are indicated and labeled with the name of the corresponding siRNA.

Sixty-four JEV genome sequences of the four genotypes available in GenBank (including 34 Chinese strains) were aligned using the SeqMan program. siRNAs against the eight selected genes were obtained using the miRNA design algorithm from Invitrogen. Thirteen siRNAs whose targets showed ≥95% conservation among the 34 Chinese strains and ≥80% conservation among the 64 strains (Figure 1 and Table 1) were selected and cloned into pcDNA 6.2–GW/EmGFP-miR according to the manufacturer's recommendations (the sequences of hairpin siRNA inserts were shown in Table S1). As genotypes I and III are the predominant genotypes in the Chinese mainland [11], these 13 selected siRNA targets were highly conserved in genotypes I and III and reasonably well conserved in genotypes II and IV. Thus the 13 selected siRNAs were designed to be fully complementary to the viral RNA of genotypes I and III, and somewhat imperfectly complementary to the viral RNA of genotypes II and IV.

**Table 1:** Target sequences of the siRNAs.

siRNA name	Target sequence (all sequences are from 5′ to 3′)
PrM-54	CATTGCAGACGTTATCGTGAT
NS1-447	TTGGAACAGCATGCAAATCGA
NS1-473	TCGGCTTTGGCATCACATCAA
NS1-630	GAAACTTGAGAGGGCAGTCTT
NS1-1207	TACACTGATTTGGCGAGGTAT
NS2A-193	ATCCTGAATGCCGCCGCTATA
NS2A-461	GACTAATGGTCTGCAACCCAA
NS2B-123	GTCCTACGTGGTGTCAGGAAA
NS3-1112	AGATTGCAATGTGCCTCCAAA
NS3-1469	CAGAGGCAAAGATCATGTTAG
NS4A-289	GCAGAGGTTCCTGGAACCAAA
NS4B-115	ACTGATGTGCCTGAACTGGAA
NS5-741	AGTGTGGAGAGGGCCAAAGTA
NC	GTTTCCGAGGCCATAAGTATT

**Figure 2:**
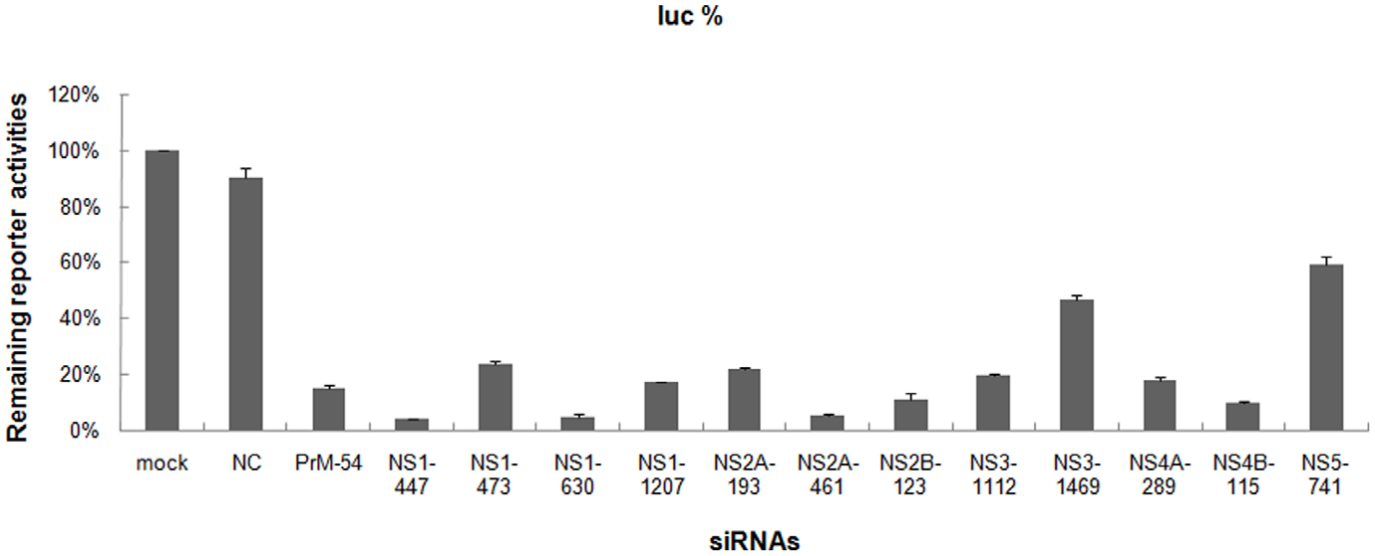
siRNA efficacy validation. BHK-21 cells were transfected with 400 ng of siRNA-encoding pcDNA 6.2–GW/EmGFP-miR vector, 170 ng of the corresponding siQuant reporter vector and 17 ng of internal control vector pRL-TK. Cells were harvested 48 hours post-transfection, and the luciferase activities were detected. Data represent the average values from triplicate experiments.

The siRNA-encoding pcDNA 6.2–GW/EmGFP-miR vectors or the negative control (NC) were co-transfected together with the corresponding siQuant vectors into Baby hamster kidney cell line (BHK-21; ATCC, USA) cells. Using the Dual-Luciferase Reporter Assay system, we selected nine out of 13 siRNAs that could knockdown the expression of the corresponding targets by greater than 80% compared with the mock control (transfected subcloned siQuant vector without the siRNA-encoding pcDNA 6.2–GW/EmGFP-miR vectors) and the NC (*P*<0.01) (Figure 2).

### Comparison of the inhibitory effect on target expression between single siRNAs and combinations of multiple siRNAs from single miRNA-like polycistrons

**Figure 3:**
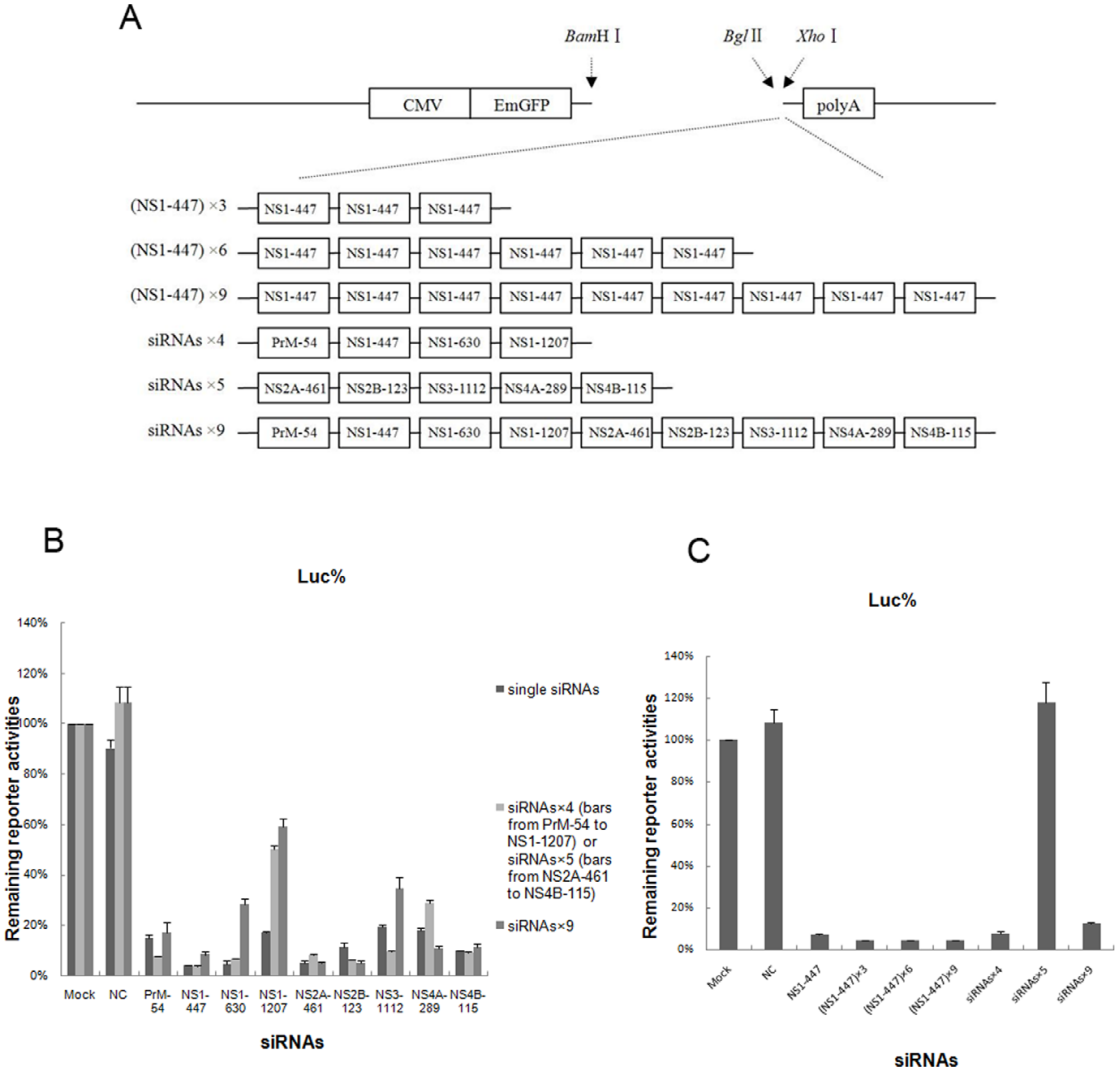
Comparison of the inhibitory effect on target expression between single siRNAs and miRNA-like polycistrons. (A) Schematic representations of the pcDNA 6.2–GW/EmGFP-miR vectors expressing different combinations of siRNA cassettes. (B) 400 ng of siRNAs×4, siRNAs×5 or siRNAs×9 was co-transfected into BHK-21 cells along with 170 ng of each of the corresponding siQuant reporters and 17 ng of the internal control pRL-TK. Cells were harvested 48 hours post-transfection and the luciferase activities were detected. (C) 400 ng of NS1-447, (NS1-447)×3, (NS1-447)×6, (NS1-447)×9, siRNAs×4, siRNAs×5 or siRNAs×9 was co-transfected into BHK-21 cells along with 170 ng of each of the corresponding siQuant reporters and 17 ng of the internal control pRL-TK. Cells were harvested 48 hours post-transfection and the luciferase activities were detected. Data represent the average values from triplicate experiments.

Using the clone strategy described in the Materials and Methods section, we obtained six miRNA-like polycistron constructs expressing different combinations of siRNAs (Figure 3A). NS1-447, the most effective siRNA, was multiplied to generate (NS1-447)×3, (NS1-447)×6, and (NS1-447)×9. siRNAs×4 were constructed from four of the nine siRNAs (PrM-54, NS1-447, NS1-630, and NS1-1207), and siRNAs×5 were formed from the other five siRNAs (NS2A-461, NS2B-123, NS3-1112, NS4A-289, and NS4B-115). All nine of the siRNAs were constructed into a chain to generate siRNAs×9.

The miRNA-like polycistron vectors or the NC were co-transfected together with the corresponding siQuant vectors into BHK-21 cells. Using the Dual-Luciferase Reporter Assay system, the inhibitory effects of miRNA-like polycistrons on the expression of each target were compared with single siRNAs (Figure 3B, 3C). Figure 3B showed that the expression of each target could also be inhibited efficiently by miRNA-like polycistrons (siRNAs×4, siRNAs×5, and siRNAs×9). Although the inhibitory efficacy of each siRNA fluctuated when chained as polycistrons, the efficacies of most siRNAs could also maintain a high level, and the efficacies of some siRNAs were even enhanced when chained in polycistrons. As shown in Figure 3C, as the number of copies of NS1-447 increased from 1 to 3 to 6 to 9, the expression of its target decreased from 7.1% to 4.3% to 4.0% to 4.2%, respectively, compared with the mock control (*P*<0.01). The efficacy of NS1-447 expressed from siRNAs×4 and siRNAs×9 was similar to that of the single copy of NS1-447. As a negative control, siRNA×5 could not inhibit the expression of the target because it did not contain NS1-447.

### Inhibitory effects on JEV strain SA 14-14-2 replication of the transient transfection of single siRNAs or combinations of multiple siRNAs from single miRNA-like polycistrons

**Figure 4:**
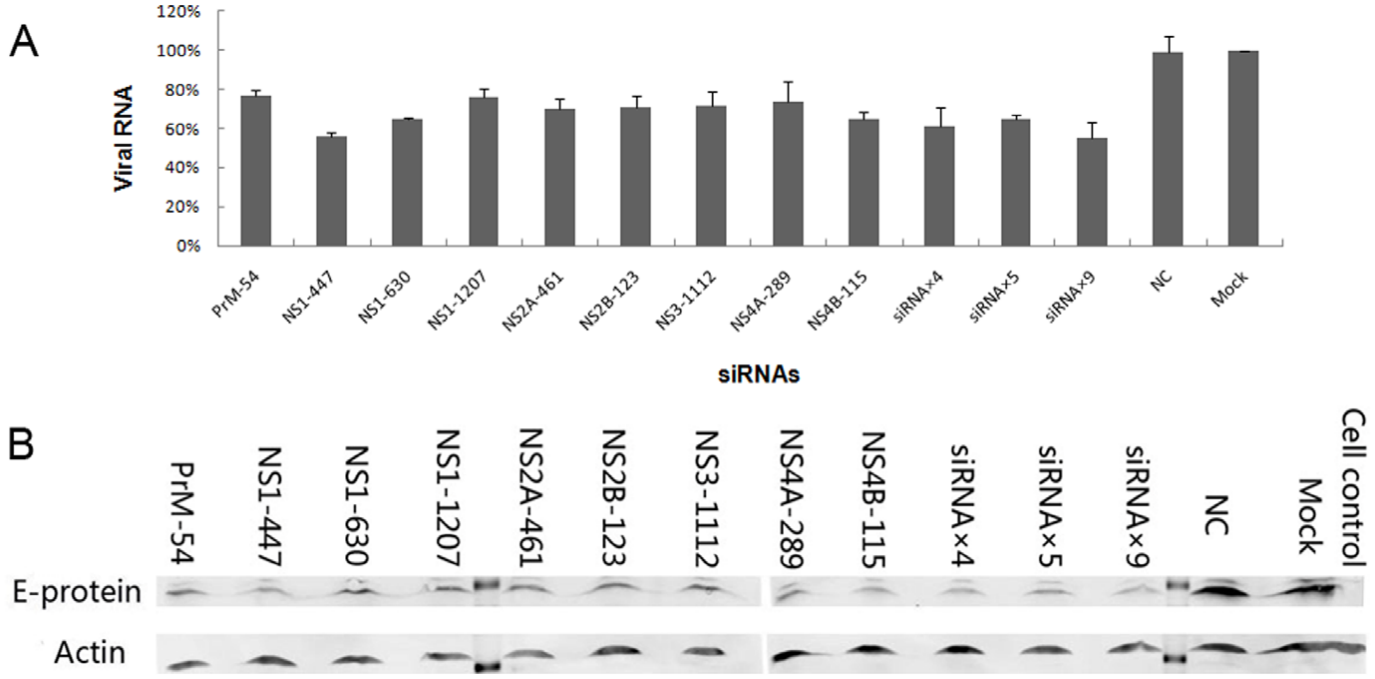
Inhibitory effects on JEV strain SA 14-14-2 replication by transient transfection of siRNA-encoding vectors. Cells seeded in 24-well plates were transfected with 800 ng of single siRNAs or miRNA-like polycistrons. Twenty-four hours post-transfection, the cells were infected with JEV strain SA 14-14-2 at a MOI of 0.1. (A) Comparison of the number of viral RNA copies at 24 h post-infection among the transfected cells. Data represent the average values from triplicate experiments. (B) Western blot analysis of gene suppression at the protein level at 24 h post-infection. E protein was detected as the target and Actin was detected as the internal control. The relative quantities of E protein compared with the mock control from lane PrM-54 to lane siRNAs×9 were 38.7%, 29.2%, 45.2%, 43.8%, 44.1%, 46.6%, 42.4%, 37.5%, 39.0%, 32.9%, 33.7% and 31.9%.

To test if the nine selected siRNAs or miRNA-like polycistrons could inhibit efficiently the replication of JEV in infected cells, BHK-21 cells were challenged with JEV strain SA 14-14-2 at a MOI of 0.1 after being transiently transfected with siRNA-encoding pcDNA 6.2–GW/EmGFP-miR vectors for 24 h. The inhibitory effects of siRNAs on JEV replication were analyzed at the RNA and protein levels by real-time RT-PCR and western blot analysis. Twenty-four hours post-infection, viral RNA copies in cultured cells were determined by real-time RT-PCR from each well. The copy number of virus RNA from siRNA-transfected cells dropped to 55.5%–77.1% compared with the mock control (non-transfected BHK-21 cells) (*P*<0.05) (Figure 4A). NS1-447 was the most effective single siRNA, and siRNAs×9 was the most effective miRNA-like polycistron. Twenty-four hours post-infection, the E (envelope) protein of JEV expressed in BHK-21 cells was detected by mouse anti-JEV E-D3 monoclonal antibody, and actin protein was detected as an internal control. E protein expression in siRNA-transfected cells reduced to 29.2%–46.6% compared with the mock control (Figure 4B). Similarly, NS1-447 was the most effective single siRNA, and siRNAs×9 was the most effective miRNA-like polycistron.

To analyze the transfection efficiency of the siRNA-encoding pcDNA 6.2–GW/EmGFP-miR vector transiently transfected by Lipofectamine 2000, the single siRNA-encoding pcDNA 6.2–GW/EmGFP-miR transfected BHK-21 cells were assayed by flow cytometry to calculate the percentage of EmGFP positive cells. As shown in Figure S1, only 32.9% of the cells were EmGFP positive. This result indicated that the low siRNA transfection efficiencies reduced the efficacy of our siRNA constructs, and that reasonable anti-JEV effects could be obtained even at low siRNA transfection efficiencies.

### Inhibitory effects on JEV strain SA 14-14-2 replication by stable cell lines constitutively expressing siRNAs

To minimize the variables associated with transient transfection, we constructed four stable cell lines constitutively expressing NS1-447, (NS1-447)×6, siRNAs×9, and the NC. NS1-447 was the most effective single siRNA, (NS1-447)×6 was the hexamer of NS1-447 and siRNAs×9 was the most effective polycistron of all nine siRNAs.

**Figure 5:**
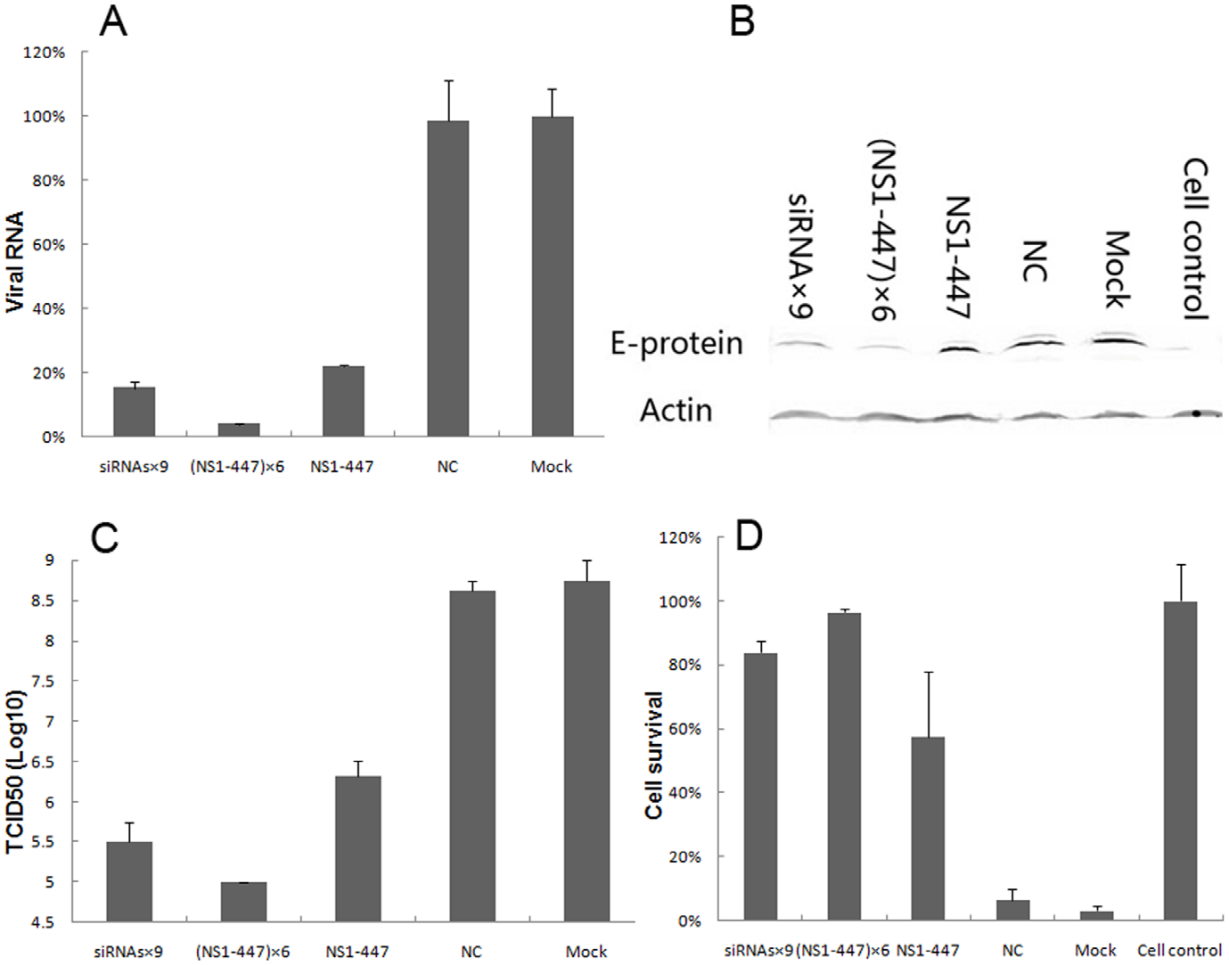
Inhibitory effects on JEV strain SA 14-14-2 replication by stable cell lines constitutively expressing siRNAs. Stable cell lines were seeded in 24-well or 96-well plates and were infected with JEV strain SA 14-14-2 at a MOI of 0.1 when the cell monolayer reached 90–100% confluence. (A) Comparison of the number of viral RNA copies at 24 h post-infection. (B) Western blot analysis of gene suppression at the protein level at 36 h post-infection, E protein was detected as the target and Actin was detected as the internal control. (C) Comparison of the viral titers in culture supernatants at 48 h post-infection. (D) Comparison of cell viability using the MTS assay at 96 h post-infection.

The four stable cell lines or normal BHK-21 cells were seeded in 24-well or 96-well plates at equal cell numbers. When the cell layer reached 90–100% confluence, the cells were infected with JEV strain SA 14-14-2 at a MOI of 0.1. Twenty-four hours post-infection, viral RNA copies in cultured cells were determined by real-time RT-PCR from each well. The copy number of viral RNA from stable lines constitutively expressing siRNAs×9, (NS1-447)×6 and NS1-447 dropped significantly to 15.1%, 3.9% and 21.8%, respectively, compared with the mock control (infected BHK-21 cells) (*P*<0.01) (Figure 5A). Thirty-six hours post-infection, E protein expression was determined by western blot analysis. E protein expression in stable cell lines constitutively expressing siRNAs×9, (NS1-447)×6 and NS1-447 was significantly reduced compared with the mock control and the NC (Figure 5B). Forty-eight hours post-infection, viral titers in the culture supernatants were examined for each well. Viral titers from stable cell lines dropped significantly to 5–6.32 TCID_50_/0.1 ml, compared with a viral titer in the mock control of 8.75 TCID_50_/0.1 ml (*P*<0.01) (Figure 5C). Ninety-six hours post-infection, the morphological changes in the stable cell lines were examined by phase-contrast microscopy, and cell viability assays were performed to analyze the proliferation conditions of each stable cell line. In the stable cell lines constitutively expressing siRNAs×9, (NS1-447)×6 and NS1-447, most cells were alive compared with the cell control (non-infected BHK-21 cells), and approximately 95% of cells were killed in the mock control and the NC (*P*<0.01) (Figure 5D and Figure S2). The results of viral RNA and E protein analysis, and the results of viral titer and cell viability assays, indicated that (NS1-447)×6 and siRNAs×9 were the two most effective agents at inhibiting the replication of JEV strain SA 14-14-2.

### Comparison of the inhibitory effects of miRNA-like polycistrons on genotypes I and III of JEV

To test if the miRNA-like polycistrons, (NS1-447)×6 and siRNAs×9, that were shown to inhibit the live attenuated vaccine strain SA14-14-2, could also inhibit the replication of the two major epidemic genotypes of JEV (genotypes I and III), three wild type strains (SH53, SH101 and SA14) of genotypes I and III, which are predominant in the Chinese mainland, were used to infect cells containing the two microRNA-like polycistrons. The nine selected siRNAs were designed to be fully complementary to the viral RNA of genotypes I and III because all target sequences of the nine selected siRNAs were extremely conserved in genotypes I and III.

**Figure 6:**
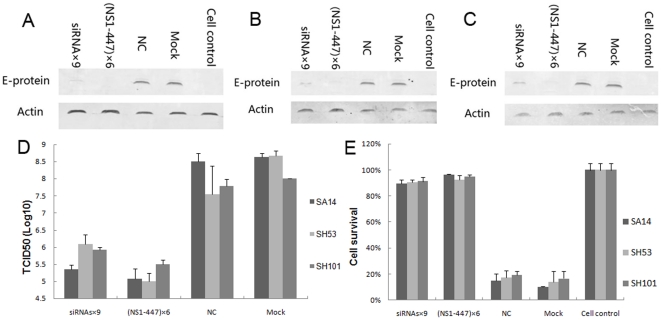
Comparison of the inhibitory effects of miRNA-like polycistrons on JEV genotype I and III strains. Stable cell lines or normal BHK-21 cells seeded in 24-well or 96-well plates were infected with JEV strain SA14, SH53 or SH101 at a MOI of 0.1. (A) Western blot analysis of E protein expression of strain SA14 at 36 h post-infection. (B) Western blot analysis of E protein expression of strain SH53 at 36 h post-infection. (C) Western blot analysis of E protein expression of strain SH101 at 36 h post-infection. (D) Comparison of viral titers in the culture supernatants at 48 h post-infection. (E) Comparison of cell viability using the MTS assay at 96 h post-infection.

The four stable cell lines or normal BHK-21 cells were seeded in 24-well or 96-well plates. When the cell layer reached 90–100% confluence, the cells were infected with JEV strains SA 14, SH53 or SH101 at a MOI of 0.1. Thirty-six hours post-infection, E protein expression was detected by western blot analysis. Compared with the mock control and the NC, E protein expression from each of the strains (SA14, SH53 and SH101) was significantly inhibited in the stably trasfected cell lines constitutively expressing (NS1-447)×6 and siRNAs×9 (Figure 6A, 6B, 6C). Forty-eight hours post-infection, viral titers in the culture supernatants were examined. The viral titers of SA14, SH53 and SH101 dropped drastically compared with the mock control and the NC (Figure 6D). Ninety-six hours post-infection, cell viability assays were performed to analyze the proliferation ability of the stable cell lines. In the (NS1-447)×6 and siRNAs×9 stably-expressing cell lines, most of the cells infected with SA14, SH53, and SH101 were alive compared with the mock control and the NC, in which over 80% of cells were killed (Figure 6E). The results of viral E protein analysis, viral titers and cell viability assays indicated that (NS1-447)×6 and siRNAs×9 were also effective against the two major epidemic genotypes of JEV, genotypes I and III.

### Prediction of the inhibitory efficacy of miRNA-like polycistrons on genotype II and genotype IV of JEV

To confirm that siRNAs×9 that inhibited the replication of JEV genotypes I and III could also inhibit the replication of the two endemic genotypes of JEV (genotypes II and IV), the target sequences of the latter genotypes, containing some nucleotide mismatches with their cognate siRNAs, were synthesized and cloned into the siQuant vector (Table 2), using the Dual-Luciferase siRNA-to-target reporter assay system. Then, the inhibitory efficacy of siRNAs×9 on the mutated target sequences was examined. Compared with the inhibitory efficacy of siRNAs×9 on perfectly complementary target sequences shown in Table 2, the inhibitory efficacy of siRNAs×9 on mutated target sequences was attenuated to various degrees, depending on the nucleotide mismatches within the target regions. The inhibitory efficacy was governed by the position and the quantity of the mismatched base pairs. When single nucleotide mismatches occurred near the 5′ or 3′ ends of the target sequences, siRNAs×9 could also inhibit the expression of the target. However, when the nucleotide mismatches occurred in the middle of the target sequences or multiple nucleotide mismatches occurred simultaneously, the inhibitory effect of siRNAs×9 was significantly reduced or even eliminated (Table 2). In general, the inhibitory efficacy shown for the mutated target sequences of genotypes II and IV indicated that siRNAs×9 could theoretically inhibit the replication of JEV genotypes II and IV.

**Table 2:** Schematic representations of the nucleotide differences in the target sequences between genotypes II and IV and the targets we selected.

Target types	Target sequences (all sequence from 5′ to 3′)	Remaining reporter activities (compared with the mock control)
PrM-54 target	CATTGCAGACGTTATCGTGAT	16.8%
Genotype II	---C--G-----C--A--T--	97.8%
Genotype IV	---C--G--T--C--------	96.3%
NS1-447 target	TTGGAACAGCATGCAAATCGA	8.3%
Genotype II	C--------------------	19.7%
Genotype IV	C--------T--------T--	95.2%
NS1-630 target	GAAACTTGAGAGGGCAGTCTT	28.3%
Genotype II	---------------T--T--	72.0%
Genotype IV	---G-----------C-----	90.8%
NS1-1207 target	TACACTGATTTGGCGAGGTAT	59.3%
Genotype II	-----------------A---	68.0%
Genotype IV	---------C----------C	98.8%
NS2A-461 target	GACTAATGGTCTGCAACCCAA	4.7%
Genotype II	---------C---------G-	88.6%
Genotype IV	----C----------------	38.6%
NS2B-123 target	GTCCTACGTGGTGTCAGGAAA	5.1%
Genotype II	---T-----------------	37.9%
Genotype IV	---T-----------------	37.9%
NS3-1112 target	AGATTGCAATGTGCCTCCAAA	34.5%
Genotype II	-------------------G-	79.5%
Genotype IV	-A-----TG-T--------G-	100.0%
NS4A-289 target	GCAGAGGTTCCTGGAACCAAA	11.0%
Genotype II	-----------C-----T---	100.0%
Genotype IV	-----A--GT-A--------G	91.2%
NS4B-115 target	ACTGATGTGCCTGAACTGGAA	11.3%
Genotype II	-----------A---------	98.6%
Genotype IV	---------------------	11.3%

The inhibitory efficacies of siRNAs×9 on each target were estimated using the Dual-Luciferase siRNA-to-target reporter assay system.

## Discussion

JEV is classified into four genotypes depending on the nucleotide sequence of its genomic RNA, with a fifth genotype being postulated. Genotype III was the first genotype to be described, and is the most widely distributed [10], followed by genotype I, which is also an epidemic genotype of JEV. Genotypes I and III are the predominant genotypes in the mainland of China [11]. Genotypes II and IV are endemic genotypes. Strain Muar, isolated in 1952, may represent the fifth genotype [10]. Previous anti-JEV studies focused on genotype III of JEV and showed that siRNA-induced gene silence could inhibit efficiently the replication of one JEV strain of genotype III (Nakayama strain, or SA 14-14-2 strain) in cultured cells [3], [25], [27]. However, these effective siRNAs have only been confirmed in one strain of genotype III and may not be suitable for other strains because of the nucleotide sequence divergence among genotype III strains. Furthermore, these siRNAs confirmed in genotype III could not provide sufficient protection from infection by other JEV genotypes because of the higher genetic variability between the four (or possibly five) genotypes of JEV [1], [4], [8], [9], [10], [11]. In addition, one or two siRNAs are not sufficient to prevent RNAi-resistant escape mutants of this virus [28], [36].

Previous studies showed that using siRNA expression vectors based on miR-30, miR-155, miR-106b or miR-17-92, could achieve RNA interference of multiple genes [22], [29], [30], [31], [32], [33]. In these studies, the regions of pri-miRNAs corresponding to mature miRNAs were replaced with heterologous stems to express siRNAs without compromising their silencing activity. Just like most miRNAs that are transcribed from polycistronic pri-miRNAs, a reconstructed vector could also express two or more different siRNAs from a single transcript for effective inhibition of two or more different target mRNAs. This platform has been applied successfully in anti-HIV research to prevent the escape mutants of persistent HIV infection [22], [29].

In this study, considering the molecular epidemiological characteristics and the geographic distribution of the four genotypes of JEV, the 13 selected siRNA targets were highly conserved in genotypes I and III and reasonably well conserved in genotypes II and IV. In theory, these siRNAs would be effective against all four genotypes of JEV. These 13 siRNAs were constructed into the pcDNA 6.2–GW/EmGFP-miR vector based on the miR-155 backbone. The nine siRNAs found to inhibit the expression of their corresponding targets by greater than 80% in the Dual-Luciferase assay system, were selected, and their inhibitory effects on JEV replication were tested. The inhibitory effects of various combinations of multiple siRNAs from single polycistrons were also tested. Our miRNA-like polycistron constructs containing four, five or nine different siRNAs showed good inhibitory effects on the expression of each target, and the efficacy of some siRNAs were even increased when chained into polycistrons, compared with the vectors expressing single siRNAs. This is the first study to describe the application of long chain miRNA-like polycistrons (with up to nine different siRNAs) without significantly compromising silencing activity. miRNA-like polycistron constructs containing three, six and nine copies of the same siRNA cassette showed no significant changes in silencing activity. As the number of copies of NS1-447 increased from 1 to 3, 6 and 9, the expression of its target decreased from 7.1% to 4.3%, 4.0% and 4.2%, respectively. This indicated that the appropriate number of siRNA copies would provide maximum silencing activity, and that there is no need to increase persistently the copy number of siRNAs. This finding differed from that of a previous study in which, using the miR-155 backbone, inhibition of the reporter increased along with the number of siRNA copies, from 2 to 4 to 8 [30]. Another related study showed that, although each addition of siRNA to the transcript resulted in an increase of the total amount of mature siRNA generated, the addition of siRNA copies did not increase significantly the inhibitory efficacy [37]. These differences may be the result of the different lengths of the vectors, the different types of processing of each siRNA and the different saturated concentrations of siRNAs.

Because of the importance of genotype III in China, and in Asia as a whole, JEV genotype III strain SA 14-14-2 was used first to test the inhibitory effects of siRNAs. Judging by the inhibitory effect of the viral RNA and E protein 24 h post-infection, the transient transfection of each siRNA-encoding vector and miRNA-like polycistron construct could inhibit the replication of strain SA 14-14-2 to a certain extent. Low transfection efficiency may greatly limit the anti-JEV effects of siRNAs. To minimize the negative impact of low transfection efficiency, we constructed four stable cell lines constructively expressing three representative siRNA transcripts and the NC to further validate the inhibitory effects. From the results of viral RNA and E protein analyses, viral titers and cell viability assays, we confirmed that the selected siRNAs could effectively inhibit the replication of strain SA 14-14-2. A single siRNA, NS1-447, provided effective inhibition. Multimerization of NS1-447 up to six copies maximized the inhibitory effect. siRNAs×9 was less effective than (NS1-447)×6, because all other eight siRNAs were less effective than NS1-447. Although less effective than (NS1-447)×6, multimerization of nine different siRNAs in a single transcript also increased significantly the inhibitory effect compared with a single copy of NS1-447. These results indicated that the single siRNA-encoding vectors and miRNA-like polycistron constructs selected in the Dual-Luciferase siRNA-to-target reporter assay system could inhibit significantly the replication of JEV strain SA 14-14-2, provided the delivery efficiency of siRNA-encoding vectors was sufficiently high. Indeed, delivery of siRNA is one of the most challenging problems in the application of RNAi in modern medicine. Many local and systemic delivery methods for siRNAs or siRNA-encoding plasmids, such as lipid-based delivery, viral vector-based delivery, cell-penetrating peptide-based delivery, and poly (lactic-co-glycolic acid) (PLGA) nanoparticle-based delivery have been reported in the past few years [26], [38], [39], [40], [41], but there is no generalized delivery approach for RNAi, with different disease targets requiring different delivery strategies. For JEV, a rabies virus glycoprotein (RVG) has been used successfully to specifically deliver anti-JEV siRNAs into neuronal cells across the blood-brain barrier [26]. Despite various efforts, further work should be conducted in vivo to investigate the most appropriate siRNA delivery strategies.

The applicability of (NS1-447)×6 and siRNAs×9 in the inhibition of JEV genotypes I and III was validated by testing three wild type strains of genotypes I and III, SH53, SH101, and SA14. Both of the miRNA-like polycistron constructs could significantly inhibit the replication of the three JEV strains, with (NS1-447)×6 being slightly more effective than siRNAs×9. This result indicated that (NS1-447)×6 and siRNAs×9 have potential applicability in treating acute viral encephalitis caused by genotypes I and III of JEV. Divergence and genetic variability within the two genotypes could be tolerated by the two miRNA-like polycistrons. Considering the fact that RNAi-resistant escape mutants within target regions may be generated following siRNA treatment [36], (NS1-447)×6 may be less effective than siRNAs×9, since it only has a single target. siRNAs×9 may therefore be more suitable for broad-spectrum therapy, alleviating the negative impact of escape mutants.

Unfortunately, JEV strains of genotypes II and IV were unavailable to validate the applicability of the two miRNA-like polycistrons in this study, because these two genotypes are rare in mainland China. However, we designed an alternative method to validate the inhibitory effects of the two miRNA-like polycistrons on genotype II and IV strains. After analyzing the genome sequences of genotypes II and IV available in the GenBank database, the target sequences of genotypes II and IV which contain mutations were synthesized and cloned into the reporter plasmid, siQuant. The inhibitory effects of siRNAs×9 on each mutated target were then estimated (Table 2), and the inhibitory effect of (NS1-447)×6 on the mutated targets of NS1-447 were estimated (data not shown). In agreement with previous studies [42], [43], we found that perfect matches in the central region of the target is critical for the effective inhibition of RNAi, and single mismatches occurring in the central region could eliminate the inhibitory effect of RNAi. In addition, multiple nucleotide mismatches in one target could eliminate the inhibitory effect of siRNA. Conversely, single mismatches occurring near the 5′ or 3′ ends of the target sequence could be well tolerated. In summary, the position and quantity of mismatches between the target sequence and the cognate siRNA affect the efficiency of inhibition. Although mutated nucleotides could attenuate the inhibitory effect of siRNAs×9 to varying degrees, the effect of extending the siRNA chain to siRNAs×9 could be complementary in preventing the inefficacy of two or three pre-designed siRNAs. Furthermore, the copy number (or proportion) of each siRNA in the chain could be adjusted according to the genotype of target JEV strain to achieve maximal inhibitory effect. This reporter system demonstrated, in theory, the inhibitory effect of siRNAs×9 on genotype II and IV strains of JEV. (NS1-447)×6 would also be effective at inhibiting genotype II strains because single mutated nucleotides at the 5′ end of the target would not affect the efficiency of RNAi. In contrast, (NS1-447)×6 would not be suitable for inhibiting genotype IV strains, because single mutated nucleotides in the centre of the target would completely eliminate silencing activity.

In conclusion, the results of this study offer the possibility of inhibiting the replication of JEV strains of the four genotypes using a single miRNA polycistron containing multiple siRNAs. The strong, broad-spectrum, anti-JEV activity of the miRNA polycistron could be applied to the development of effective antiviral gene therapies.

## Materials and Methods

### Cell cultures and virus assays

Baby hamster kidney cell line (BHK-21; ATCC, USA) cells were propagated and maintained in Dulbecco's modified Eagle's medium (DMEM; Gibco, Invitrogen, USA) supplemented with 10% fetal bovine serum (FBS; Gibco, Invitrogen, USA) at 37°C with 5% CO_2_.

The JEV strains SA 14-14-2, SA 14, SH101 and SH53 were provided by Dr. Yongxin Yu, Department of First Viral Vaccine, National Institute for the Control of Pharmaceutical and Biological Products. Strains SA 14-14-2 and SA 14 belong to genotype III. Strains SH101 and SH53 belong to genotype I. All viruses were propagated in BHK-21 cells. The 50% tissue culture infective dose (TCID_50_) was calculated in BHK-21 cells using 96-well plates and the Reed-Muench formula [44].

### Plasmid construction

For siRNA-encoding plasmids, the highly conserved regions of eight genes (PrM, NS1, NS2A, NS2B, NS3, NS4A, NS4B and NS5) among the four genotypes of JEV were determined by sequence alignment using the SeqMan program available within the Lasergene 7 package (DNAStar, USA). Thirteen siRNAs based on these conserved regions (Figure 1 and Table 1) were designed using the miRNA design algorithm (http://rnaidesigner.invitrogen.com/rnaiexpress/setOption.do?designOption=mirna&pid=509133211138749536). These 13 siRNAs were individually cloned into pcDNA 6.2–GW/EmGFP-miR (Invitrogen, USA), a RNA polymerase II-driven siRNA expression vector based on the miR-155 backbone, according to the manufacturer's recommendations (the sequences of hairpin siRNA inserts were shown in Table S1). Combination of multiple siRNAs cassettes from a single miRNA-like polycistron construct was performed by digesting a single siRNA hairpin unit with *Bam*HI and *Xho*I and inserting the digested fragment into the *Bgl*II/*Xho*I sites of pcDNA 6.2–GW/EmGFP-miR. We obtained different combinations of multiple siRNAs by repeating this procedure. We simultaneously designed a negative control (NC), which comprised scrambled siRNA containing the same nucleotide component as PrM-54. Every clone was verified by PCR and sequencing.

To construct the reporter vector, the target sequences of these 13 siRNAs (Table 1) were cloned into the *Bgl*II/*Apa*I sites of the siQuant vector (Genordia AB, Sweden), as described previously, to fuse the target sequences to the *firefly* luciferase gene [45].

### siRNA efficacy validation

BHK-21 cells were plated in 24-well plates (0.5 ml medium/well) and cultured at 37°C with 5% CO_2_ for 24 h. When the cell layer reached 60–70% confluence, siRNA-encoding pcDNA 6.2–GW/EmGFP-miR vectors (400 ng), as well as the reporter plasmids (170 ng of target-encoding siQuant vectors and 17 ng of pRL-TK as an internal control) were co-transfected using Lipofectamine 2000 (Invitrogen, USA) according to the manufacturer's recommendations. Forty-eight hours post-transfection, cells were harvested and the *firefly* luciferase and *Renilla* luciferase activities were measured on SpectraMax M5 (Molecular Devices, USA) using the Dual-Luciferase Reporter Assay System (Promega, USA). The inhibitory effects generated by the siRNA-eocoding pcDNA 6.2–GW/EmGFP-miR vectors were expressed as normalized ratios between the reporter and control luciferase activities [31], [42], [45].

### Transfection and virus infection

BHK-21 cells were seeded in 24-well plates. When the cell layer reached 60–70% confluence, each siRNA-encoding pcDNA 6.2–GW/EmGFP-miR vector (800 ng) was transiently transfected into the cells. Twenty-four hours post-transfection, the cells were infected with JEV strain SA 14-14-2 at a multiplicity of infection (MOI) of 0.1. After 1–2 h adsorption, the inocula were removed and cells were incubated in DMEM supplemented with 10% FBS.

### Real-time reverse transcription (RT)-PCR

The following primers: forward, 5′-CCTCCGTCACCATGCCAGTCTTAG-3′ and reverse, 5′-TTCGCCATGGTCTTTTTCCTCTCG-3′, were used to amplify the region spanning nt 3976–4108 in the genome of JEV strain SA 14-14-2. This region was then cloned into the pGEM-T Easy Vector (Promega, USA) to construct the standard plasmid. Using the standard plasmid we obtained a standard curve.

The RNAeasy Mini kit (Qiagen, Germany) was used for RNA extraction from every well 24 h post-infection according to the manufacturer's instructions. A reverse transcription (RT) reaction was carried out using Superscript III Reverse Transcriptase (Invitrogen, USA) in a 20 µl reaction mixture with 1.2 µg of total RNA. Real-time PCR was conducted using an ABI Prism 7000 Real-time PCR system (Applied Biosystems, USA). Reactions were performed in a 50 µl volume that contained 2 µl of cDNA, 1 µl of each primer and 25 µl of Power SYBR Green PCR Master Mix (Applied Biosystems, USA). Viral RNA was quantified using the standard curve.

### SDS-PAGE and Western blot analysis

BHK-21 cells at 24 h or 36 h post-infection were washed twice with PBS and lysed with 100 µl M-PER mammalian protein extraction reagent (Pierce, USA). Then, 20 µl lysates were boiled for 5–10 min after mixed with 5×loading buffer. Proteins were separated by 12% sodium dodecylsulfate polyacrylamide gel electropheresis (SDS-PAGE) and transferred to nitrocellulose membrane (Bio-Rad, USA) using a semi-dry electrophoretic cell (Bio-Rad, USA). For immunoblotting, membranes were first incubated with mouse anti-JEV E-D3 monoclonal antibody (Beijing Protein Innovation, China) at a dilution of 1∶2000 or mouse anti-actin monoclonal antibody (Sigma, USA) at a dilution of 1∶5000. Membranes were then incubated with IRDye 680 conjugated goat anti-mouse secondary antibody (Li-Cor Biosciences, USA) at a dilution of 1∶5000 or 1∶15000. Protein bands were detected and quantified using the Li-Cor Odyssey system and the Odyssey infrared imaging software (Li-Cor Biosciences, USA).

### Flow cytometry assay

At 48 h post-transfection, the siRNA-encoding pcDNA 6.2–GW/EmGFP-miR transfected cells and the control cells were washed twice with PBS, trypsinized and resuspended in PBS. EmGFP positive cells were evaluated by flow cytometry using a FACSCanto II flow cytometer (BD Bioscience, USA).

### Establishment of stable cell lines constitutively expressing siRNAs

BHK-21 cells were seeded in 24-well plates. When the cell layer reached 50% confluence, the selected siRNA-encoding pcDNA 6.2–GW/EmGFP-miR vector (400 ng) was transiently transfected into cells. Twenty-four hours post-transfection, cells cultured in DMEM containing 4 µg/ml blasticidin were trypsinized and replated into 10 cm tissue culture plates. The medium was replaced with fresh DMEM containing blasticidin (4 µg/ml) every 3–4 days until blasticidin-resistant colonies were obtained. Individual colonies were obtained by serial dilution and were screened by cell viability assays following JEV infection. Established clones that could maximally inhibit the propagation of virus were propagated and maintained in DMEM containing 2 µg/ml blasticidin.

### Cell viability assay

BHK-21 cells constitutively expressing siRNAs or control cells were seeded in 96-well plates for 24 h and then infected with JEV at a MOI of 0.1. Ninety-six hours post-infection, 20 µl MTS/PMS (Promega, USA) was added to each well and the absorbance at 490 nm was measured according to the manufacturer's recommendations.

### Statistical analysis

All experiments were repeated at least thrice, and standard deviation of the mean was determined. Statistical analyses were performed by paired *t*-test using Statistical Product and Service Solutions (SPSS, USA).

## Supporting Information

Figure S1 **Flow cytometryanalysis of the transfectionefficiency of the siRNA-encoding vector.** Cells seeded in 24-well plates were transfectedwith 800 ngof single siRNAs. Twenty-four hours post-transfection, the cells were infected with JEV strain SA 14-14-2 at a MOI of 0.1. At 48 h post-transfection, EmGFPpositive cells were evaluated by flow cytometry. Control-P1, non-transfectedBHK-21 cells; Sample-P1, single siRNA-encoding pcDNA6.2–GW/EmGFP-miRtransfectedBHK-21 cells.
(TIF)

Figure S2 **Inhibitory effects on JEV strain SA 14-14-2 replication by stable cell lines constitutively expressing siRNAs.** Stable cell lines were seeded in 24-well or 96-well plates and were infected with JEV strain SA 14-14-2 at a MOI of 0.1 when the cell layer reached 90–100% confluence. Morphological changes in the stable cell lines or normal BHK-21 cells at 96 h post-infection.
(TIF)

Table S1 **The sequences of hairpin siRNA inserts of pcDNA 6.2–GW/EmGFP-miR.**
(DOC)
