# Supplementary figures and images for: Broad-Spectrum Antiviral Activity of RNA Interference against Four Genotypes of Japanese Encephalitis Virus Based on Single MicroRNA Polycistrons
Source: PLoS One. 2011 Oct 18;6(10):e26304. doi: 10.1371/journal.pone.0026304 (PMC3196537; doi:10.1371/journal.pone.0026304)

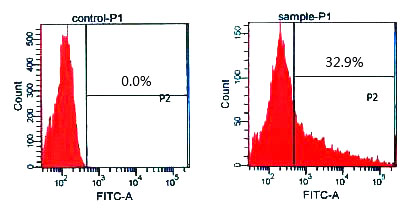

Supplement: Figure S1 — Flow cytometryanalysis of the transfectionefficiency of the siRNA-encoding vector. Cells seeded in 24-well plates were transfectedwith 800 ngof single siRNAs. Twenty-four hours post-transfection, the cells were infected with JEV strain SA 14-14-2 at a MOI of 0.1. At 48 h post-transfection, EmGFPpositive cells were evaluated by flow cytometry. Control-P1, non-transfectedBHK-21 cells; Sample-P1, single siRNA-encoding pcDNA6.2–GW/EmGFP-miRtransfectedBHK-21 cells. (TIF) [file pone.0026304.s001.tif]

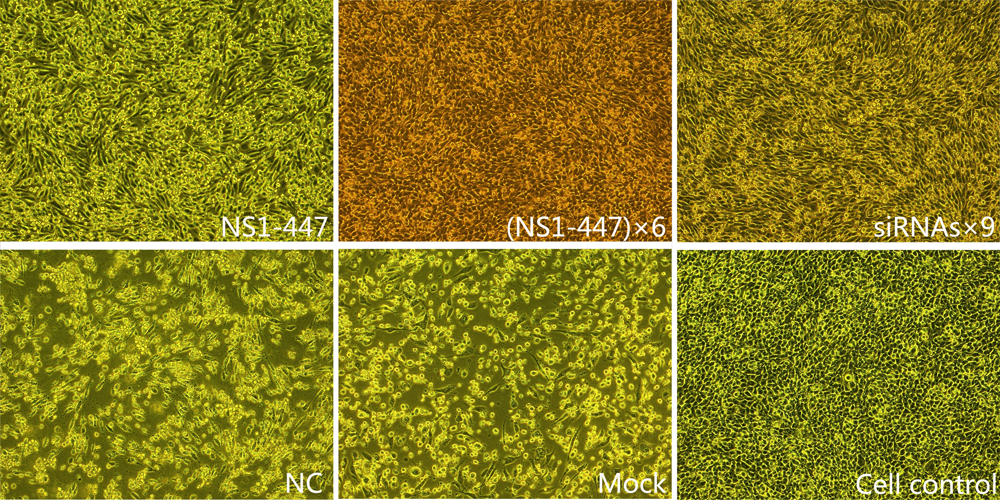

Supplement: Figure S2 — Inhibitory effects on JEV strain SA 14-14-2 replication by stable cell lines constitutively expressing siRNAs. Stable cell lines were seeded in 24-well or 96-well plates and were infected with JEV strain SA 14-14-2 at a MOI of 0.1 when the cell layer reached 90–100% confluence. Morphological changes in the stable cell lines or normal BHK-21 cells at 96 h post-infection. (TIF) [file pone.0026304.s002.tif]
