# Supplementary material for: Broad-Spectrum Antiviral Activity of RNA Interference against Four Genotypes of Japanese Encephalitis Virus Based on Single MicroRNA Polycistrons
Source: PLoS One. 2011 Oct 18;6(10):e26304. doi: 10.1371/journal.pone.0026304 (PMC3196537; doi:10.1371/journal.pone.0026304)
Supplement: Table S1 — The sequences of hairpin siRNA inserts of pcDNA 6.2–GW/EmGFP-miR. (DOC) [file pone.0026304.s003.doc]

| Oligonucleotide | Sequences (5’-3’) |
| --- | --- |
| PrM_54_top | TGCTGATCACGATAACGTCTGCAATGGTTTTGGCCACTGACTGACCATTGCAGGTTATCGTGAT |
| PrM_54_bottom | CCTGATCACGATAACCTGCAATGGTCAGTCAGTGGCCAAAACCATTGCAGACGTTATCGTGATC |
| NS1_447_top | TGCTGTCGATTTGCATGCTGTTCCAAGTTTTGGCCACTGACTGACTTGGAACAATGCAAATCGA |
| NS1_447_bottom | CCTGTCGATTTGCATTGTTCCAAGTCAGTCAGTGGCCAAAACTTGGAACAGCATGCAAATCGAC |
| NS1_473_top | TGCTGTTGATGTGATGCCAAAGCCGAGTTTTGGCCACTGACTGACTCGGCTTTCATCACATCAA |
| NS1_473_bottom | CCTGTTGATGTGATGAAAGCCGAGTCAGTCAGTGGCCAAAACTCGGCTTTGGCATCACATCAAC |
| NS1_630_top | TGCTGAAGACTGCCCTCTCAAGTTTCGTTTTGGCCACTGACTGACGAAACTTGAGGGCAGTCTT |
| NS1_630_bottom | CCTGAAGACTGCCCTCAAGTTTCGTCAGTCAGTGGCCAAAACGAAACTTGAGAGGGCAGTCTTC |
| NS1_1207_top | TGCTGATACCTCGCCAAATCAGTGTAGTTTTGGCCACTGACTGACTACACTGATGGCGAGGTAT |
| NS1_1207_bottom | CCTGATACCTCGCCATCAGTGTAGTCAGTCAGTGGCCAAAACTACACTGATTTGGCGAGGTATC |
| NS2A_193_top | TGCTGTATAGCGGCGGCATTCAGGATGTTTTGGCCACTGACTGACATCCTGAACCGCCGCTATA |
| NS2A_193_bottom | CCTGTATAGCGGCGGTTCAGGATGTCAGTCAGTGGCCAAAACATCCTGAATGCCGCCGCTATAC |
| NS2A_461_top | TGCTGTTGGGTTGCAGACCATTAGTCGTTTTGGCCACTGACTGACGACTAATGCTGCAACCCAA |
| NS2A_461_bottom | CCTGTTGGGTTGCAGCATTAGTCGTCAGTCAGTGGCCAAAACGACTAATGGTCTGCAACCCAAC |
| NS2B_123_top | TGCTGTTTCCTGACACCACGTAGGACGTTTTGGCCACTGACTGACGTCCTACGGTGTCAGGAAA |
| NS2B_123_bottom | CCTGTTTCCTGACACCGTAGGACGTCAGTCAGTGGCCAAAACGTCCTACGTGGTGTCAGGAAAC |
| NS3_1112_top | TGCTGTTTGGAGGCACATTGCAATCTGTTTTGGCCACTGACTGACAGATTGCAGTGCCTCCAAA |
| NS3_1112_bottom | CCTGTTTGGAGGCACTGCAATCTGTCAGTCAGTGGCCAAAACAGATTGCAATGTGCCTCCAAAC |
| NS3_1469_top | TGCTGCTAACATGATCTTTGCCTCTGGTTTTGGCCACTGACTGACCAGAGGCAGATCATGTTAG |
| NS3_1469_bottom | CCTGCTAACATGATCTGCCTCTGGTCAGTCAGTGGCCAAAACCAGAGGCAAAGATCATGTTAGC |
| NS4A_289_top | TGCTGTTTGGTTCCAGGAACCTCTGCGTTTTGGCCACTGACTGACGCAGAGGTCTGGAACCAAA |
| NS4A_289_bottom | CCTGTTTGGTTCCAGACCTCTGCGTCAGTCAGTGGCCAAAACGCAGAGGTTCCTGGAACCAAAC |
| NS4B_115_top | TGCTGTTCCAGTTCAGGCACATCAGTGTTTTGGCCACTGACTGACACTGATGTCTGAACTGGAA |
| NS4B_115_bottom | CCTGTTCCAGTTCAGACATCAGTGTCAGTCAGTGGCCAAAACACTGATGTGCCTGAACTGGAAC |
| NS5_741_top | TGCTGTACTTTGGCCCTCTCCACACTGTTTTGGCCACTGACTGACAGTGTGGAGGGCCAAAGTA |
| NS5_741_bottom | CCTGTACTTTGGCCCTCCACACTGTCAGTCAGTGGCCAAAACAGTGTGGAGAGGGCCAAAGTAC |
| NC_top | TGCTGAATACTTATGGCCTCGGAAACGTTTTGGCCACTGACTGACGTTTCCGACCATAAGTATT |
| NC_bottom | CCTGAATACTTATGGTCGGAAACGTCAGTCAGTGGCCAAAACGTTTCCGAGGCCATAAGTATTC |
